# 2-aminopurine suppresses the TGF-β1-induced epithelial–mesenchymal transition and attenuates bleomycin-induced pulmonary fibrosis

**DOI:** 10.1038/s41420-017-0016-3

**Published:** 2018-02-13

**Authors:** Dong Weng, Jian-xia Chen, Hao-hao Li, Feng Liu, Li-dan Zhou, Hai-peng Liu, Rui-juan Zheng, Yan Jiang, Zhong-hua Liu, Baoxue Ge

**Affiliations:** 1 Clinical Translation Research Center, Shanghai Pulmonary Hospital, Tongji University School of Medicine, Shanghai, China; 2 Shanghai TB Key Labotatory, Shanghai Pulmonary Hospital, Tongji University School of Medicine, Shanghai, China; 3 Department of Microbiology and Immunology, Tongji University School of Medicine, Shanghai, China; 4 Present Address: Department of Respiratory Medicine, Shanghai Pulmonary Hospital, Tongji University School of Medicine, Shanghai, China

## Abstract

The epithelial–mesenchymal transition (EMT) is a multifunctional cell process involved in the pathogenesis of numerous conditions, including fibrosis and cancer. Idiopathic pulmonary fibrosis (IPF) is a progressive and fatal disease characterized by fibroblast accumulation and collagen deposition in the lungs. The fibroblasts involved in this process partially originate from lung epithelial cells via the EMT. Evidence suggests that the EMT contributes to progression, invasion, and metastasis of various types of cancer. We screened a series of 80 compounds for the ability to interfere with the EMT and potentially be applied as a therapeutic for IPF and/or lung cancer. We identified 2-aminopurine (2-AP), a fluorescent analog of guanosine and adenosine, as a candidate in this screen. Herein, we demonstrate that 2-AP can restore E-cadherin expression and inhibit fibronectin and vimentin expression in TGF-β1–treated A549 lung cancer cells. Moreover, 2-AP can inhibit TGF-β1-induced metastasis of A549 cells. This compound significantly attenuated bleomycin (BLM)-induced pulmonary inflammation, the EMT, and fibrosis. In addition, 2-AP treatment significantly decreased mortality in a mouse model of pulmonary fibrosis. Collectively, we determined that 2-AP could inhibit metastasis in vitro by suppressing the TGF-β1-induced EMT and could attenuate BLM-induced pulmonary fibrosis in vivo. Results of this study suggest that 2-AP may have utility as a treatment for lung cancer and pulmonary fibrosis.

## Introduction

The epithelial–mesenchymal transition (EMT) is a multifunctional cell process characterized by loss of cell polarity, loss of cell–cell adhesion, detachment from the basal lamina, cytoskeletal rearrangement, and migration into the provisional matrix^1^. These vast phenotypic shifts are accompanied by significant changes in molecular expression and signal transduction within cells, including downregulation of E-cadherin (a biomarker of epithelial cells) and upregulation of fibronectin and vimentin (biomarkers of mesenchymal cells)^2^. To ascertain the occurrence of the EMT, the following molecular hallmarks are commonly assessed: increased nuclear localization of β-catenin; increased expression of N-cadherin, fibronectin, vimentin, and Snail1/2; and decreased production of E-cadherin, desmoplakin, cytokeratin, and occludin^3^.

TGF-β1 is a potent inducer of the EMT that is common to Smad and non-Smad signaling pathways. TGF-β1 signaling directly activates expression of the EMT-associated transcription factors Snail, Slug, and Twist1^4^. A transcriptional complex comprising Smad3/Smad4 and Snail can bind to regulatory promoter sequences of the genes that encode the epithelial-junction proteins E-cadherin and occludin. This results in TGF-β1–mediated repression of gene expression^5^. In addition, TGF-β1 regulates the expression of matrix metalloproteinases (MMPs), such as MMP2 and MMP9, and of components of the extracellular matrix, such as fibronectin and collagens, by activating EMT-associated transcription factors^6^. TGF-β1-induced non-Smad signaling also promotes the EMT. Specifically, TGF-β1 activates the PI3K/Akt/mTOR pathway, thereby producing increased protein synthesis, motility, and cell invasion during the EMT by means of mTOR complex 1^7^. Other growth factors also can synergize with TGF-β1 signaling to facilitate the EMT; these include epidermal growth factor and fibroblast growth factor, which act through receptor tyrosine kinases and are released into the epithelial cell microenvironment^8^.

The EMT is crucial for in the pathogenesis of many conditions, including fibrosis and cancer. Idiopathic pulmonary fibrosis (IPF) is a chronic and often lethal disease that involves progressive scarring, or fibrosis, of the lungs. Patients with IPF have a mean survival time of 2–3 years from diagnosis^9^. In the United States, ~50,000 new cases of IPF are diagnosed each year, and as many as 40,000 Americans die from IPF annually^9^. Despite efforts to understand IPF, the etiology and epidemiology of this condition have not been elucidated fully. The excessive fibrosis in IPF is attributable, at least in part, to enhanced formation and survival of fibroblasts. These cells are the primary source of extracellular matrix proteins, such as fibronectin and collagens^10^. Accumulation of extracellular matrix proteins leads to irreversible destruction of the lung parenchyma and a progressive decline in lung function^11^. Because fibroblast expansion and concomitant deposition of collagen fibrils are key processes in IPF, much attention has been given to understanding molecular mechanisms that lead to fibroblast proliferation and synthesis of matrix proteins. In addition to proliferation of lung-resident fibroblasts, fibrogenic fibroblasts originate from lung epithelial cells via the EMT^12, 13^.

Lung cancer is relatively common and is the leading cause of death among patients with cancer. Approximately 90% of lung cancer deaths are attributable to metastasis, a complex process whereby cancer cells spread from a primary site and form tumors at distant sites. Evidence suggests that the EMT contributes to progression, invasion, and metastasis of various types of cancer^2, 14, 15^.

In this study, we aimed to identify compounds that could inhibit the EMT, pulmonary fibrosis, and lung cancer metastasis. We determined that 2-aminopurine (2-AP) could inhibit the EMT, induced by TGF-β1, in A549 cells. Moreover, 2-AP can significantly attenuate inflammation, the EMT, and fibrosis in a mouse model of bleomycin (BLM)-induced pulmonary fibrosis. Hence, 2-AP may have utility as treatment for pulmonary fibrosis or lung cancer.

## Results

### Identification of compounds that suppress the TGF-β1-induced EMT

**Fig. 1:**
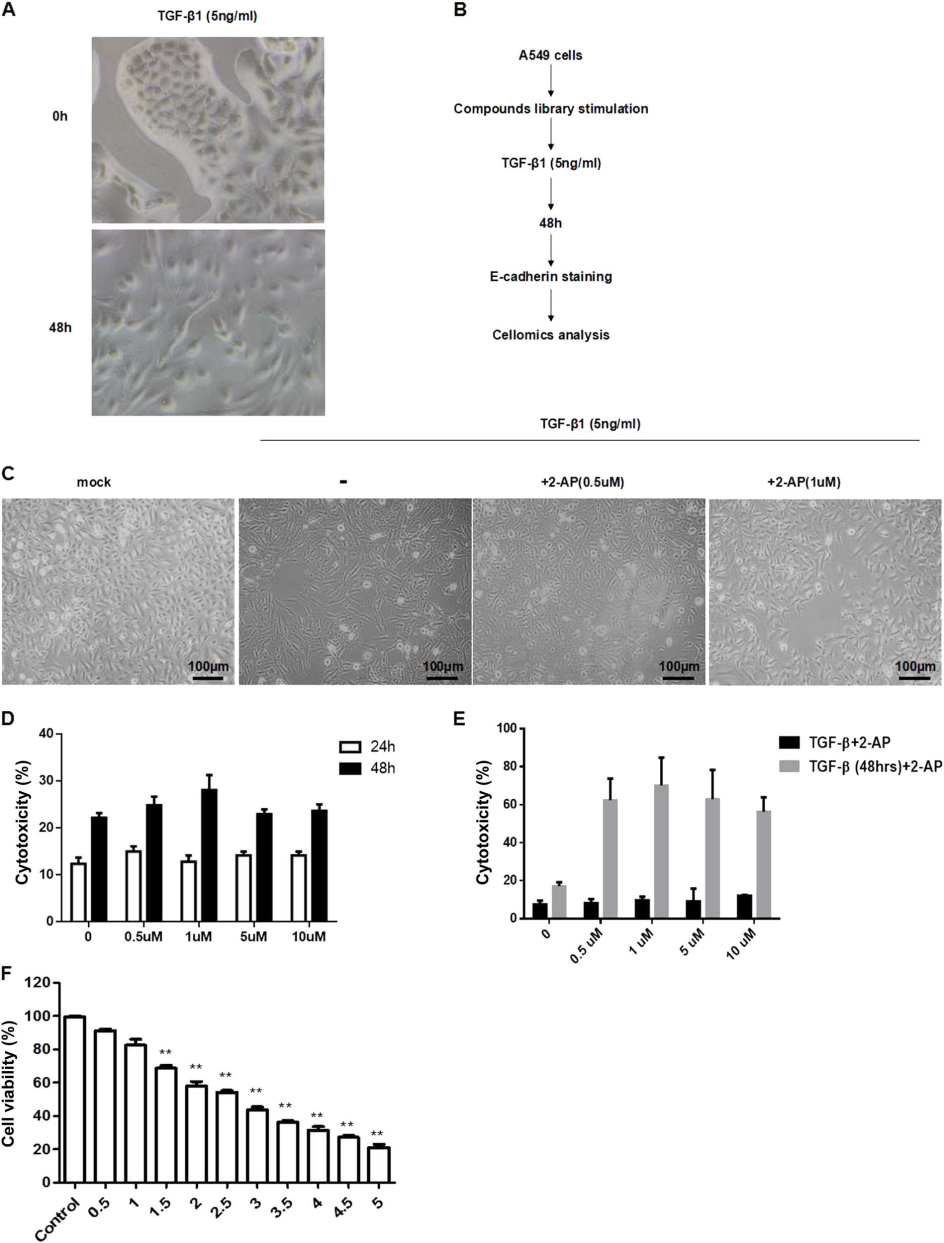
Identification of compounds that regulate the TGF-β1-induced EMT. **a** A549 cells were incubated with TGF-β1 (5 ng/mL) for 48 h, and the mean fluorescence intensity was determined by cellomics analysis. **P < 0.01. **b** Compounds were screened according to the procedure shown; the mean fluorescence intensity was detected by cellomics analysis. **c** A549 cells were treated with 2-AP (0.5 or 1 µM) and TGF-β1 (5 ng/mL), and morphologic changes were observed. **d** The effects of 2-AP on cell toxicity were determined by the LDH assay. A549 cells were treated with 2-AP at concentrations of 0 (DMSO was used as a control), 0.5, 1, 5, and 10 µM for 24 or 48 h. **e** The effects of 2-AP on cell toxicity in the context of TGF-β1 stimulation were determined with the LDH assay. A549 cells were treated with 2-AP at concentrations of 0 (DMSO was used as a control), 0.5, 1, 5, and 10 µM for 48 h; 2-AP was delivered either simultaneously with TGF-β1 (black bars) or 48 h after TGF-β1 stimulation (gray bars). **f** The effects of 2-AP on cell viability were determined by the MTT assay in A549 cells stimulated with 5 ng/mL of TGF-β1. Cells also were treated with 2-AP at concentrations of 0 (DMSO was used as a control), 0.5, 1, 1.5, 2, 2.5, 3, 3.5, 4, 4.5, or 5 µM for 48 h. **P < 0.01. Values are represented as the mean ± SD of three independent experiments performed in triplicate

To develop a functional screen for efficient inhibitors of the EMT, we carried out a cellomics analysis. A549 cells were cultured in 96-well plates (5000 cells per well) and were treated for 1 h with 10 different compounds in a final concentration of 10 nM. Cells then were stimulated with TGF-β1 in a final concentration of 5 ng/mL. A549 cells displayed stellate and elongated fibroblast-like morphologies after TGF-β1 stimulation (Fig. 1a). Two days after TGF-β1 stimulation, A549 cells were labeled with mouse anti-human E-cadherin IgG and anti-mouse IgG conjugated with Alexa 488. The mean fluorescence intensity was monitored and quantified with a cellomics scan reader (Fig. 1b). To test the ability of TGF-β1 to induce the EMT and to ascertain the sensitivity of E-cadherin fluorescence to indicate the EMT, samples exposed to TGF-β1 only also were evaluated. Our findings indicated that the cellomics assay was sufficiently sensitive to detect inhibitors of the EMT.

Applying this screen, we narrowed the series of compounds to 10 potential inhibitors of the EMT and ultimately determined that only 2-AP showed significant, reliable rescue of E-cadherin downregulation induced by TGF-β1 (Supplementary Fig. 1). We also found that 2-AP could reverse changes in cell morphology that occurred after TGF-β1 treatment (Fig. 1c). Subsequently, we assessed whether 2-AP was cytotoxic by means of the lactate dehydrogenase (LDH) assay. We determined that 2-AP caused little LDH release, suggesting low cytotoxicity (Fig. 1d). We also applied the LDH assay to evaluate the cytotoxicity of 2-AP in the context of TGF-β1 stimulation. We found that 2-AP posed significant cytotoxicity to mesenchymal cells (Fig. 1e). These results suggest that 2-AP has no cytotoxicity for A549 (epithelial) cells within 10 µM stimulation but kills mesenchymal cells even at a low concentration. We then assessed the effect of 2-AP on the viability of A549 cells after 48 h of exposure to 2-AP. Cells showed some loss of viability at the lowest concentration of 2-AP (0.5 µM), and there were few surviving cells after 48 h of exposure to 2-AP at the highest tested concentration of 5 µM (Fig. 1f). Hence, 2-AP might kill mesenchymal cells (Fig. 1e) but does not kill epithelial cells (Fig. 1d).

### 2-AP can inhibit TGF-β1-induced downregulation of E-cadherin and upregulation of fibronectin and vimentin

**Fig. 2:**
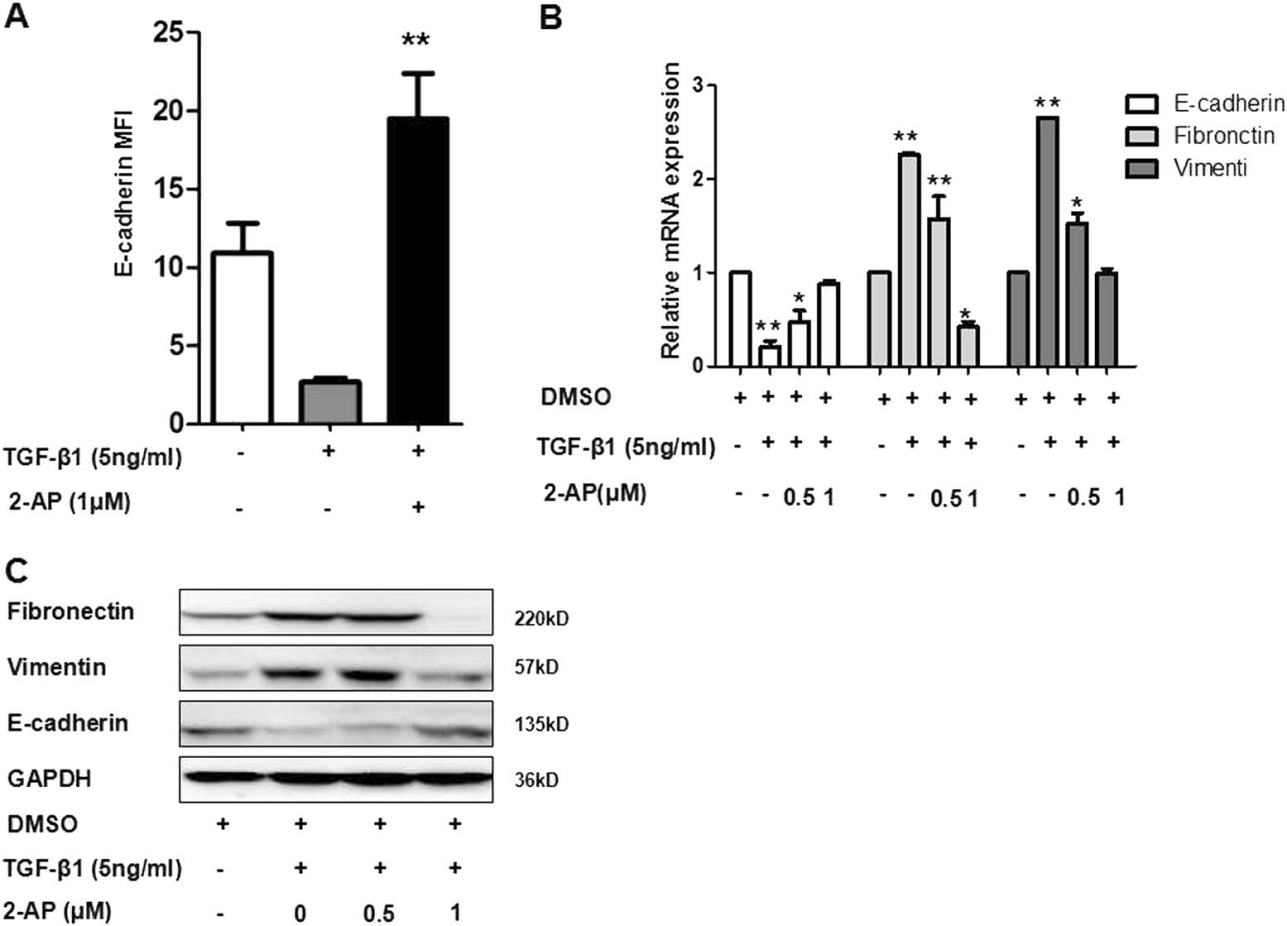
Effect of 2-AP on expression of the epithelial marker E-cadherin and the mesenchymal markers fibronectin and vimentin. **a** A549 cells were treated with 2-AP and TGF-β1 as Fig. 1c, and the mean fluorescence intensity of E-cadherin was determined. **b** A549 cells were treated with DMSO (control), 5 ng/mL of TGF-β1, 5 ng/mL of TGF-β1 + 0.5 µM of 2-AP, or 5 ng/mL of TGF-β1 + 1 µM of 2-AP for 24 h. Results of mRNA expression analysis showed between-group differences for E-cadherin, fibronectin, and vimentin. Experiments were repeated at least in triplicate (*n* = 3). *P < 0.05; **P < 0.01. **c** Western blot was applied to assess changes in protein levels of E-cadherin, fibronectin, and vimentin. GAPDH was used as an internal loading control. Data shown are representative of three independent experiments

As a confirmation of the results of our screen, we determined that 2-AP could inhibit the reduction of E-cadherin significantly (Fig. 2a). Fibronectin and vimentin are markers of mesenchymal cells (Fig. 2b), and results of real-time PCR indicated that 2-AP inhibited TGF-β1-induced downregulation of E-cadherin and upregulation of fibronectin and vimentin. The results of western blot analysis further validated that 2-AP restored expression of E-cadherin expression and inhibited TGF-β1-induced upregulation of fibronectin and vimentin (Fig. 2c).

### 2-AP can suppress expression of EMT-inducing transcription factors

**Fig. 3:**
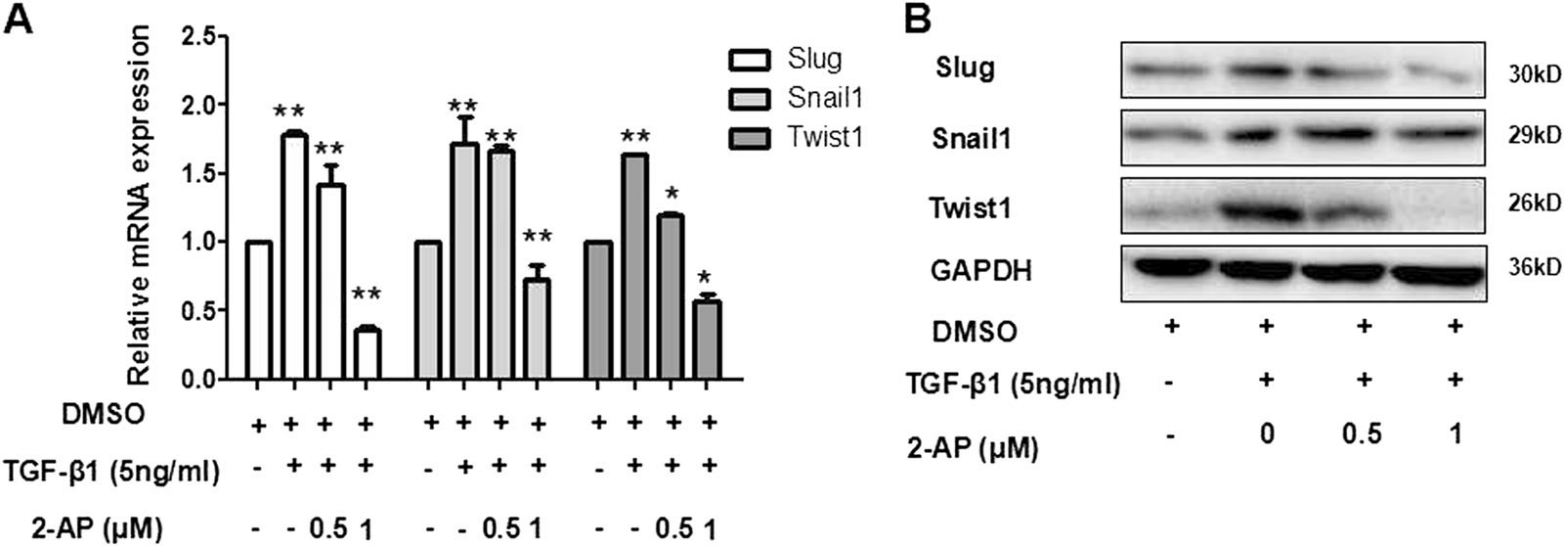
Effect of 2-AP on the EMT-inducing transcription factors Snail1, Twist1, and Slug. **a** After 24 h, differences in mRNA expression of Snail1, Twist1, and Slug were observed for the following cell treatment groups: control, 5 ng/mL TGF-β1, 5 ng/mL TGF-β1 + 2-AP (0.5 µM), and 5 ng/mL TGF-β1 + 2-AP (1 µM). Experiments were conducted at least in triplicate (*n* = 3). *P < 0.05: **P < 0.01. **b** Changes in protein expression of Snail1, Twist1, and Slug were determined by means of western blot analysis in A549 cells treated for 24 h. GAPDH was used as an internal loading control. Data shown are representative of three independent experiments

We ascertained whether 2-AP suppresses the expression of the EMT-inducing transcription factors Snail1, Twist1, and Slug using real-time PCR and western blot. We found that expression of Snail1, Slug, and Twist1 were significantly increased in TGF-β1-stimulated cells, compared with control, unstimulated cells (*P* < 0.01) (Fig. 3a, b). However, messenger RNA (mRNA) levels of Snail1, Slug, and Twist1 also were significantly inhibited by 1 µM of 2-AP (Fig. 3a). Moreover, 2-AP inhibited Slug and Twist1 protein expression and reduced the TGF-β1–associated induction of Snail1, Slug, and Twist1 expression in a dose-dependent manner.

### 2-AP can inhibit invasion and migration of A549 cells

**Fig. 4:**
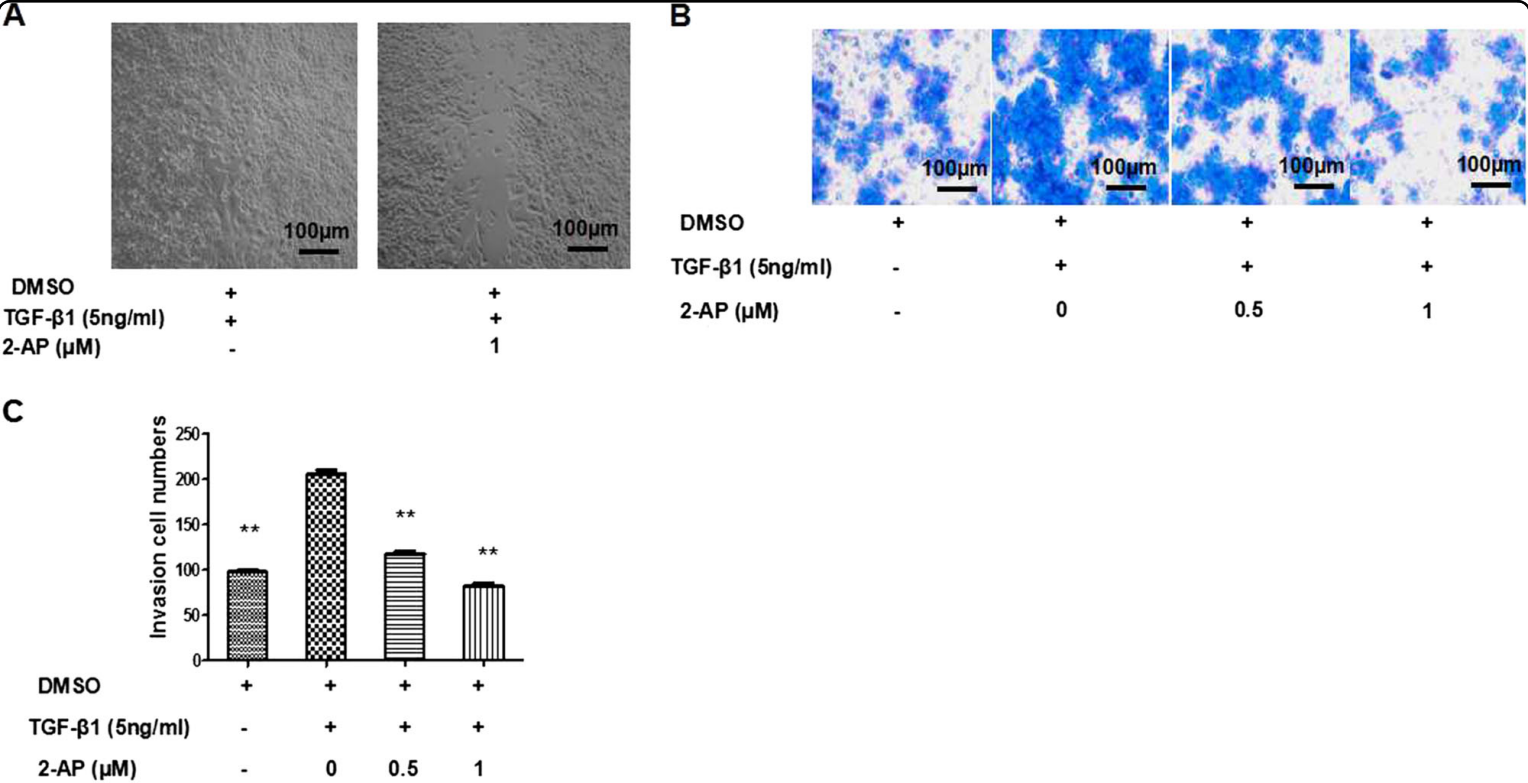
Effects of 2-AP on invasion and migration of lung cancer cells. **a** The effects of 2-AP on cell migration were determined by the wound scratch assay. Representative images are depicted. **b**, **c** The effects of 2-AP on cell invasion were determined by means of a Matrigel invasion assay, and the quantities of invading cells were determined. **P < 0.01

To explore whether 2-AP could inhibit the effects of TGF-β1-induced EMT on cell invasion and metastasis, we applied a transwell assay of migration/invasion and a Matrigel-based invasion assay. We found a significant difference in invasion for 2-AP cells that were unstimulated (control) or TGF-β1-stimulated (*P* < 0.01). We also determined that 2-AP could inhibit migration (Fig. 4a) and invasion (Fig. 4b, c) of cells stimulated with TGF-β1.

### 2-AP alleviates BLM-induced pulmonary fibrosis by inhibiting the EMT in mice

**Fig. 5:**
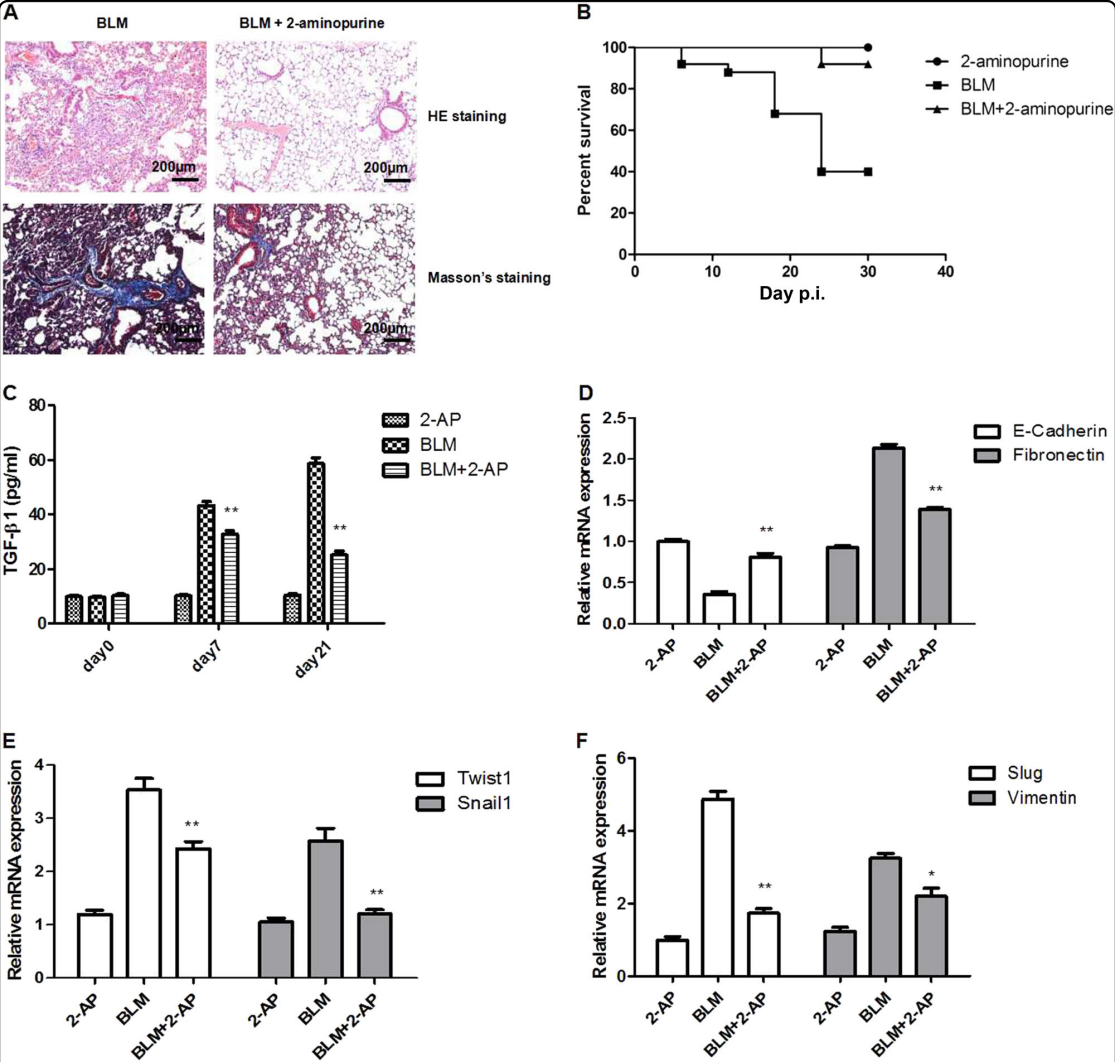
2-AP can attenuate BLM-induced pulmonary fibrosis and death in mice by inhibiting the EMT. **a** Hematoxylin-eosin (HE) and Masson’s trichrome staining of lung specimens from mice that had been treated with BLM (2.5 mg/kg) for 21 days. **b** Survival of mice treated with BLM (2.5 mg/kg) and 2-AP (2 mg/kg), as indicated (*n* = 20 per group). **c** The levels of TGF-β1 in BALF were determined by ELISA. **P < 0.01 for BLM + 2-AP vs BLM. **d** The mRNA levels of E-cadherin and fibronectin were determined in lung tissue specimens. **e** The mRNA levels of Twist1 and Snail1 in mouse lung tissues were assessed. **f** The mRNA levels of Slug and vimentin were determined in lung specimens. *P < 0.05; *P < 0.01

**Fig. 6:**
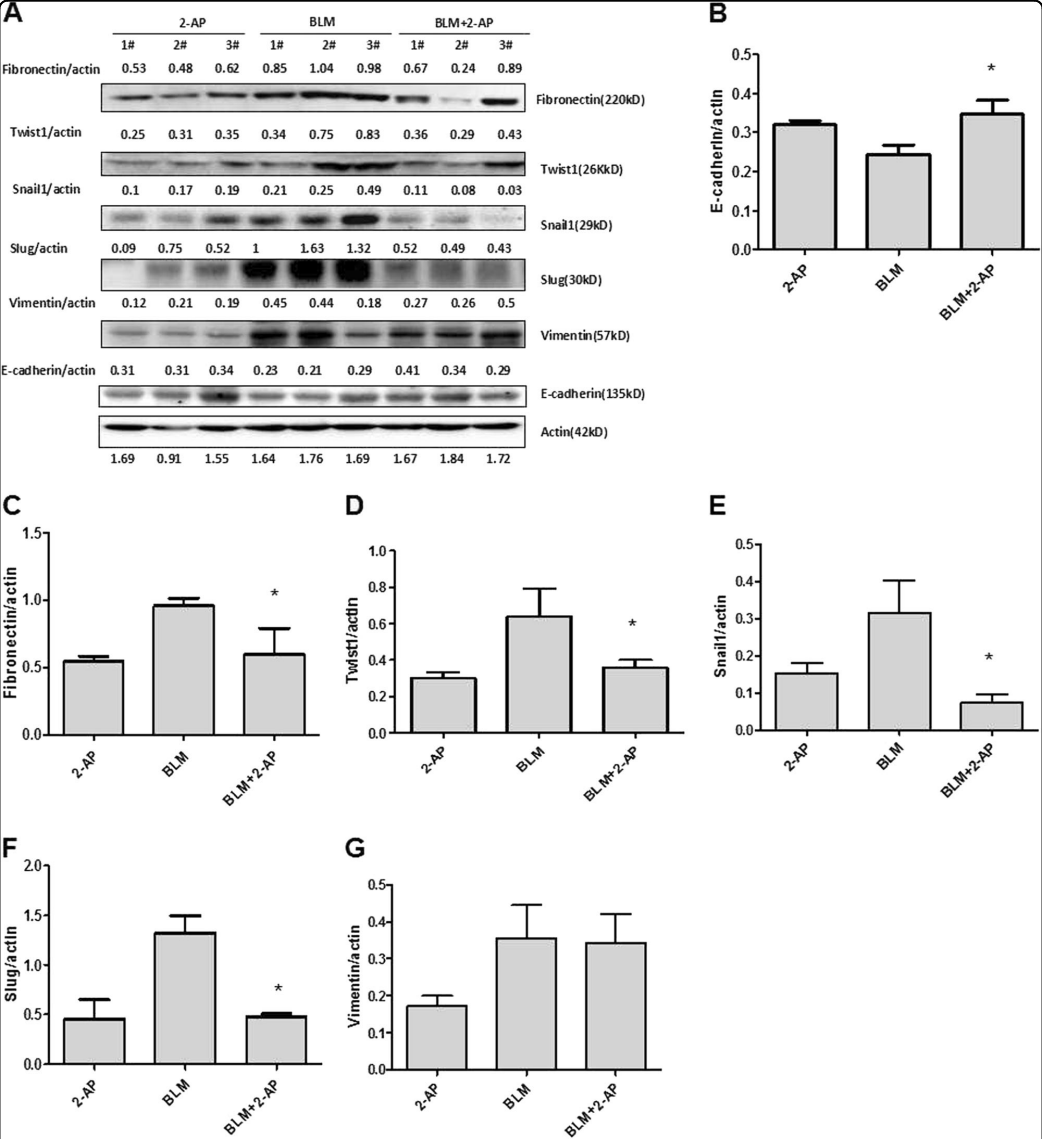
2-AP can inhibit the EMT in a mouse model of BLM-induced pulmonary fibrosis. **a** The protein expression levels of several markers of the EMT were determined by western blot analysis in mouse lung specimens (*n* = 3 per group) 21 days after stimulation with BLM. Actin was used as an internal loading control. Examined EMT markers included **b** E-cadherin, **c** fibronectin, **d** Twist1, **e** Snail1, **f** Slug, and **g** vimentin. *P < 0.05

The EMT contributes to the pathogenesis of pulmonary fibrosis. Therefore, we examined the effects of 2-AP in an animal model of BLM-induced pulmonary fibrosis. Results of histologic analysis of lung tissue indicated that 2-AP attenuated BLM-induced inflammation and fibrosis in mice (Fig. 5a). The mortality rate of mice given 2 mg/kg of 2-AP was similar to that of controls, and 2-AP significantly decreased mortality in mice that were given BLM (Fig. 5b). We then determined the level of TGF-β1 in bronchoalveolar lavage fluid (BALF) from mice. We found that 2-AP treatment resulted in significant suppression of TGF-β1 production in a model of pulmonary fibrosis (Fig. 5c). Next, we explored the effects of 2-AP on the mRNA expression of EMT markers, including E-cadherin, fibronectin, Twist1, Snail1, Slug, and vimentin in mouse lung tissue specimens. We found that 2-AP restores the expression of E-cadherin and decreases the expression of fibronectin, Twist1, Snail1, Slug, and vimentin (Fig. 5d–f). We subsequently measured the protein levels of these EMT markers in mouse lung tissue. Consistent with mRNA findings, 2-AP restored the expression of E-cadherin and decreased the protein levels of fibronectin, Twist1, Snail1, and Slug (Fig. 6a–g). These findings suggested that 2-AP might alleviate BLM-induced pulmonary fibrosis in mice by inhibiting the EMT.

## Discussion

For nearly 40 years, 2-AP has been widely used as a fluorescent molecular marker in nucleic acid research^16^. However, its physiologic function in mammals has not been investigated fully. Herein, we demonstrated that 2-AP can inhibit metastasis of lung cancer (A549) cells and can attenuate BLM-induced pulmonary inflammation and fibrosis by suppressing the TGF-β1-induced EMT. However, the mechanism by which 2-AP mediates suppression of the EMT and pulmonary fibrosis remains to be elucidated.

Many investigators have applied 2-AP as an inhibitor of PKR, a double-stranded RNA-dependent protein kinase^17^. Activation of PKR results in production of interferon (IFN)-β in the context of viral infection and yields phosphorylation of the eukaryotic translation initiation factor 2-alpha in the context of cell stress^18^. Autophosphorylation of PKR is inhibited by 2-AP; this modification is essential for PKR-mediated downstream signaling^17^. Poly (I:C), an agonist of PKR signaling, has been found to induce emphysema development and airway fibrosis^19^. In a cardiology study, PKR knockout mice were found to have little pulmonary congestion and low levels of myocardial fibrosis, compared with wild-type mice^20^. Whether 2-AP inhibits the EMT and pulmonary fibrosis by blocking PKR activation warrants further investigation.

Notably, 2-AP has been shown to inhibit the expression of lipopolysaccharide (LPS), virus-induced type I IFN-β, and the inducible isoform of nitric oxide synthase^21^. In addition, 2-AP inhibits the activation of Akt, which results in decreased nuclear translocation of phosphorylated IRF-3, a key transcription factor for IFN-β production^22^. IFN-β is a pleotropic cytokine that exhibits antiviral, anti-inflammatory, and antitumor properties by regulating the expression of numerous genes involved in crucial biologic processes, such as cell cycle progression, cell proliferation, and apoptosis^23^. Oncogenes, such as Ras and Src, can activate IFN-β signaling; in turn, IFN-β signaling has been shown to contribute to Ras-induced transformation^24^. IFN-β signaling, induced by LPS, increases the expression of SIRT1, a NAD-dependent deacetylase^25^. SIRT1 induces the EMT by cooperating with ZEB1; together, these components function to enhance cell migration and metastasis in prostate cancer. Based on these data, it seems that IFN-β is a promoter^26^. Given that 2-AP has been shown to inhibit LPS or virus-induced IFN-β production, it is plausible that 2-AP inhibits cancer cell metastasis and IPF by blocking the expression of IFN-β. More research is needed to ascertain the function of IFN-β in the TGF-β1-induced EMT and to determine whether 2-AP inhibits the EMT via IFN-β. It remains unknown whether 2-AP attenuates BLM-induced pulmonary fibrosis by directly targeting the TGF-β1-induced EMT. The cytotoxic effects of 2-AP on mesenchymal cells (i.e., fibroblasts) might partially contribute to inhibition of the EMT and fibrosis (Fig. 1e, f). We have found that 5 µM of 2-AP can inhibit cell growth (data not shown). Together, our results suggest that 2-AP might inhibit the EMT, specifically kill mesenchymal cells (i.e., fibroblasts), and inhibit cell growth (which might contribute to inhibition of the EMT and fibrosis). Work is ongoing in our laboratory to characterize these effects of 2-AP.

Taken together, we demonstrated that 2-AP negatively regulates the TGF-β1-induced EMT in A549 cells and attenuates BLM-induced pulmonary fibrosis in vivo by inhibiting the EMT. We suggest that 2-AP and its derivatives may have potential therapeutic effects for IPF and lung cancer metastasis. We intend to continue exploring these effects of 2-AP by applying more cancer cell types and by evaluating effects on metastasis in vivo.

## Materials and Methods

### Animals

Six- to 8-week-old female C57BL/6 mice, free of respiratory disease and weighing between 18 and 25 g, were kept and bred in pathogen-free conditions. All animal studies were conducted at an AAALAC-approved facility in the Laboratory Animal Center of Tongji University. Experiments were conducted in accordance with the National Institutes of Health Guide for Care and Use of Laboratory Animals, and the study was approved by the Tongji University Laboratory Animal Center, Shanghai.

### Reagents

Recombinant human TGF-β1 was purchased from R&D Systems (Minneapolis, MN). The mouse anti–E-cadherin was obtained from eBioscience (Thermo Fisher Scientific, Waltham, MA). Rabbit anti-GAPDH was from Sigma-Aldrich (St. Louis, MO). Rabbit monoclonal antibody against Slug (C19G7) was purchased from Cell Signaling Technology (Danvers, MA). Rabbit polyclonal antibody against Twist1 (L-21); mouse monoclonal antibodies against vimentin (J144), fibronectin (EP5), and Snail1 (G-7); and anti-mouse IgG were purchased from Santa Cruz Biotechnology (Santa Cruz, CA). For EMT markers in mice, we used an EMT Antibody Sampler Kit from Cell Signaling Technology. Goat anti-mouse IgG conjugated to fluorescein isothiocyanate was acquired from Invitrogen (Thermo Fisher Scientific). TGF-β1 was purchased from Peprotech (Rocky Hill, NJ). BLM, prepared by Nippon Kayaku (Tokyo, Japan), was obtained from the Pharmacy Department of Shanghai Pulmonary Hospital (China). Compound libraries were acquired from Enzo Life Sciences (Shanghai, China).

### Cell culture and drug treatment

A549 cells were cultured in Dulbecco’s Modified Eagle Media (DMEM; Invitrogen) supplemented with 10% fetal bovine serum (FBS; Hyclone, GE Healthcare Life Sciences, Logan, UT) and 1% penicillin/streptomycin. Cells were maintained in an incubator at 37 °C and 5% CO_2_, and were given fresh medium every 2 to 3 days. For cellomics screening, A549 cells were cultured in a 96-well plate (5000 cells per well) and were treated for 1 h with Screen-Well Kinase Inhibitor Library (Enzo Life Sciences). The library contains 80 known kinase inhibitors in a final concentration of 10 nM. Cells then were stimulated with TGF-β1 in a final concentration of 5 ng/mL. Two days after stimulation, A549 cells were labeled with mouse anti-human E-cadherin IgG and anti-mouse IgG conjugated with Alexa 488. The mean fluorescence intensity was monitored and quantified with an ArrayScan High-Content System (Thermo Fisher Scientific). For the MTT assay, cells were grown to 70–80% confluence in serum-free DMEM for 24 h before stimulation with TGF-β1. Cells then were incubated with 5 ng/mL of TGF-β1 and prespecified concentrations of 2-AP for 24 or 48 h. The MTT assay then was performed, according to the manufacturer’s instructions. For the LDH assay, we used A549 cells and a lactate dehydrogenase activity assay Kit (Sigma-Aldrich), according to the manufacturer’s recommendations, to ascertain the cytotoxicity of 24 or 48 h of exposure to 2-AP.

### Cell invasion and migration assays

Cell invasion was assessed with a Matrigel-based invasion assay, as specified by the manufacturer (Corning, Tewksbury, MA). Briefly, cells (1 × 10^5^ cells per well) were added to the Matrigel-embedded upper chambers, and inserts were placed into the lower chambers, which contained DMEM. Cells then were cultured for 24 h. Nonmigrating cells remaining in the upper chambers were removed and discarded, and invading cells at the bottom of the membranes were fixed with 4% formaldehyde in phosphate-buffered saline (PBS) for 10 min. Cells then were stained with 0.1% crystal violet and were counted under a microscope.

A wound healing assay was carried out under conditions of low serum (0.2% FBS). A wound was formed by scratching a confluent cell monolayer, and images were captured over time. The migration area was calculated by subtracting the scratched area from the total area of the image.

### RNA isolation and complementary DNA amplification

Total RNA was extracted from A549 cells and from mouse lung tissue using Trizol reagent, according to the manufacturer’s protocol (Takara, Shiga, Japan). RNA concentrations were determined by measuring absorption in a spectrophotometer (Nanodrop 2000, Thermo Fisher Scientific), and 1-µg aliquots of total RNA were reverse-transcribed (RT) directly using a RevertAid First Strand cDNA synthesis Kit, according to the manufacturer’s protocol (Fermentas, Thermo Fisher Scientific).

Quantitative real-time RT-PCR was performed using a SYBR Premix Ex Taq Kit (Takara) with forward and reverse primers for E-cadherin, vimentin, fibronectin, Snail1, Twist1, Slug, and GAPDH. The primers were as follows: E-cadherin forward, 5′-CCACCAAAGTCACGCTGAAT-3′ and reverse, 5′-GGAGTTGGGAAATGTGAGC-3′; vimentin forward, 5′-GAGAACTTTGCCGTTGAAGC-3′ and reverse, 5′-CTCAATGTCAAGGGCCATCT-3′; fibronectin forward, 5′-GAGCTATTCCCTGCACCTGA-3′ and reverse, 5′-CGTGCAAGGCAACCACACT-3′; Snail1 forward, 5′-CAGCGAGCTGCAGGACTCTA-3′ and reverse, 5′-GTGGGATGGCTGCCAGC-3′; Twist1 forward, 5′-TGTCCGCGTCCCACTAGC-3′ and reverse, 5′-TGTCCATTTTCTCCTTCTCTG-3′; Slug forward, 5′-TGTGTGGACTACCGCTGC-3′ and reverse, 5′-TCCGGAAAGAGGAGAGAGG-3′; GAPDH forward, 5′-GACAGTCAGCCGCATCTTC-3′ and reverse, 5′-CAACAATATCCACTTTACCAG-3′. PCR was carried out in an ABI 7500 real-time PCR System (Applied Biosystems, Foster City, CA). All samples were analyzed in triplicate.

### Western blot

The right lung of each mouse was homogenized in 200–00 µL of radioimmunoprecipitation assay (RIPA) buffer (Cell Signaling Technology), an isotonic cocktail containing protease and phosphatase inhibitors. A549 cells were cultured in 6-cm dishes and were treated with the indicated compounds for 1 h. Cells then were stimulated with TGF-β1 at a final concentration of 5 ng/mL. 2 days after stimulation, A549 cells were washed with PBS three times and were lysed by addition of RIPA cell lysis buffer with 1 mM of phenylmethylsulfonyl fluoride. After 20 min on ice, lysates were separated by centrifugation at 13,000×*g* for 5 min, and protein content was determined using a DC protein assay Kit (Bio-Rad, Hercules, CA). Samples were heated at 95 °C for 15 min and were resolved by 10% sodium dodecyl sulfate polyacrylamide gel electrophoresis for 90 min at 100 V. Samples then were transferred onto nitrocellulose membranes (Bio-Rad) at 100 V for 60 min. The membrane was preblotted in 5% milk and TBST (Tris-buffered saline, 0.1% Tween 20) for 30 min. Subsequently, the membrane was blotted with primary antibody overnight, washed, and blotted with secondary horse radish peroxidase-conjugated antibody for 60 min. Detection of bound antibody was achieved with enhanced chemiluminescence (GE Healthcare, Uppsala, Sweden).

### Model of BLM-induced pulmonary fibrosis and histopathologic analysis

Mice were injected intraperitoneally with 2-AP (2 mg/kg) or with physiologic saline and then were instilled intratracheally with BLM (2.5 mg/kg) or NaCl as control. Twenty-one days post instillation, mice were killed by decapitation, and the lungs were dissected. Lung specimens were fixed in 10% phosphate-buffered formalin, embedded in paraffin, sectioned, stained with hematoxylin-eosin solution or Masson trichrome solution, and examined for histologic changes by means of an SCN400 scanner (Leica Biosystems, Wetzlar, Germany). Survival was noted daily until day 30 after BLM challenge (*n* = 20 animals per group). Protein levels of TGF-β1 in BALF were detected by enzyme-linked immunosorbent assay (R&D Systems).

### Statistical analysis

Graphpad Prism 5 was utilized for data processing. Data are presented as mean ± standard deviation. Intergroup comparisons were made using independent sample *t* tests and one-way analysis of variance followed by the Newman–Keuls multiple comparison test. The survival curve was plotted with the Kaplan–Meier method, and survival was compared using the log-rank test. Significance was defined as *P* < 0.05.

## Supplementary information

Supplementary data
