# Supplementary material for: 2-aminopurine suppresses the TGF-β1-induced epithelial–mesenchymal transition and attenuates bleomycin-induced pulmonary fibrosis
Source: Cell Death Discov. 2018 Feb 13;4:17. doi: 10.1038/s41420-017-0016-3 (PMC5841362; doi:10.1038/s41420-017-0016-3)
Supplement: Supplementary file 1 — Supplementary data [file 41420_2017_16_MOESM1_ESM.doc]

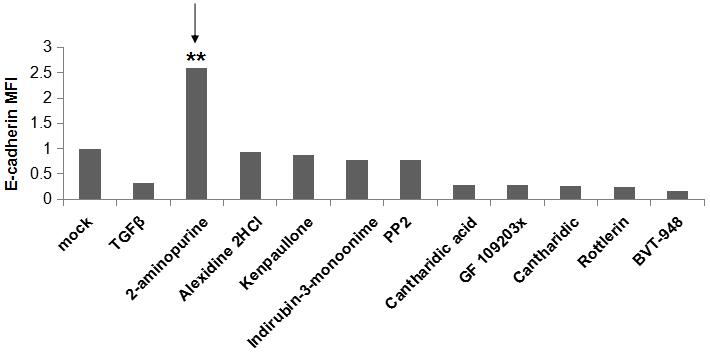


**Figure S1. Compound screening data**

A549 cells were treated with various compounds and TGF-β1 following procedure Figure 1 (B) and mean fluorescence intensity was detected by Cellomics analysis.
